# The mycoparasite *Pythium oligandrum* induces legume pathogen resistance and shapes rhizosphere microbiota without impacting mutualistic interactions

**DOI:** 10.3389/fpls.2023.1156733

**Published:** 2023-10-20

**Authors:** Maryam Hashemi, Aurélien Amiel, Mohamed Zouaoui, Kévin Adam, Hélène San Clemente, Marielle Aguilar, Rémi Pendaries, Jean-Malo Couzigou, Guillaume Marti, Elodie Gaulin, Sébastien Roy, Thomas Rey, Bernard Dumas

**Affiliations:** ^1^ Laboratoire de Recherche en Sciences Végétales, Université de Toulouse, Centre National de la Recherche Scientifique (CNRS), Université Toulouse III, Toulouse Institut National Polytechnique (INP), Auzeville-Tolosane, France; ^2^ DE SANGOSSE, Pont-Du-Casse, France; ^3^ Metatoul-AgromiX Platform, MetaboHUB, National Infrastructure of Metabolomics and Fluxomics, Toulouse, France; ^4^ AGRONUTRITION, Carbonne, France

**Keywords:** *Pythium oligandrum*, microbiota, symbiotic interaction, plant defense, legumes, isoflavonoid

## Abstract

*Pythium oligandrum* is a soil-borne oomycete associated with rhizosphere and root tissues. Its ability to enhance plant growth, stimulate plant immunity and parasitize fungal and oomycete preys has led to the development of agricultural biocontrol products. Meanwhile, the effect of *P. oligandrum* on mutualistic interactions and more generally on root microbial communities has not been investigated. Here, we developed a biological system comprising *P. oligandrum* interacting with two legume plants, *Medicago truncatula* and *Pisum sativum. P. oligandrum* activity was investigated at the transcriptomics level through an RNAseq approach, metabolomics and finally metagenomics to investigate the impact of *P. oligandrum* on root microbiota. We found that *P. oligandrum* promotes plant growth in these two species and protects them against infection by the oomycete *Aphanomyces euteiches*, a devastating legume root pathogen. In addition, *P. oligandrum* up-regulated more than 1000 genes in *M. truncatula* roots including genes involved in plant defense and notably in the biosynthesis of antimicrobial compounds and validated the enhanced production of *M. truncatula* phytoalexins, medicarpin and formononetin. Despite this activation of plant immunity, we found that root colonization by *P. oligandrum* did not impaired symbiotic interactions, promoting the formation of large and multilobed symbiotic nodules with *Ensifer meliloti* and did not negatively affect the formation of arbuscular mycorrhizal symbiosis. Finally, metagenomic analyses showed the oomycete modifies the composition of fungal and bacterial communities. Together, our results provide novel insights regarding the involvement of *P. oligandrum* in the functioning of plant root microbiota.

## Introduction

Biological control agents (BCAs) are the products based on living organisms, which address biotic stress including disease, pests, and weeds in crops (Ehlers, 2011; Shoham, 2020). BCAs can protect crops from pathogens through different mechanisms, including niche competition, production of antimicrobial compounds, or mycoparasitism (Upadhyay et al., 2021). Today, together with a heightened public awareness toward integrated pest management and sustainable agriculture, alternative disease control strategies, notably biological control, have become a rapidly growing area especially when the application of abusive chemical products threatens human health and harms the environment (Baker et al., 2020; Hashemi et al., 2021).

Among the BCAs, soil mycoparasites, such as *P. oligandrum*, gained a particular interest through their ability to parasitize fungal pathogens (Benhamou et al., 2012). *Pythium oligandrum* is a soil-borne oomycete associated with rhizosphere and root tissues and has been used in different plant systems to control various soil-borne pathogens, including Ascomycetes, Basidiomycetes, and pathogenic Oomycetes, notably, *Aphanomyces euteiches* (Daraignes et al., 2018; Bělonožníková et al., 2020; Yacoub et al., 2020; del Pilar Martínez-Diz et al., 2021). The root colonization by *P. oligandrum* is associated with induced plant growth promotion (via the production of tryptamine, an auxin precursor (Le Floch et al., 2003). *P. oligandrum* can also trigger plant immunity by releasing two major glycoproteins with elicitin activity, namely, POD-1 and POD-2, and by interplaying with iron homeostasis in roots (Takenaka et al., 2011; Bělonožníková et al., 2020; Cheng et al., 2022).

These biological activities prompted the development of *P. oligandrum* as an active ingredient of agricultural products, notably, the strain ATCC 38472. *Pythium oligandrum* ATCC 38472 was first isolated from sugar beet as an indigenous wild type strain by Dáša Veselý in 1972 in the former Czechoslovakia (now the Czech Republic) (Vesely, 1977). Then, in the 1980s, it was first applied against damping-off of sugar beet by the Slušovice cooperative as an agricultural product. Finally, in the 1990s, Dáša Veselý licensed it exclusively to the Biopreparáty company with the aim of producing Polyversum, the registered biological fungicide (Faure et al., 2020).

While the benefits of *P. oligandrum* on plant fitness and defense have been widely investigated, the impact of this mycoparasite on other types of plant–microbe interactions, notably, the mutualistic ones as well as its overall impact on microbial community in rhizosphere, was not clear. The rhizosphere is a favorable niche for the development of a wide variety of organisms, including parasitic, saprophytic, neutral, and beneficial microorganisms which can have a huge impact on plant growth and health as well as soil fertility (Compant et al., 2019; Hamid et al., 2021). Plant roots can also physically and chemically affect the rhizosphere by changing the microbial composition through providing organic carbon from the tissues or root-secreted nutrients and antimicrobials (Lemanceau et al., 2017). While the microbial community that plants recruit depends on plant genotype and agricultural management, the goal would be to optimize this microbiota to sustain plant growth and health (Lemanceau et al., 2017; Compant et al., 2019). In particular, plants establish mutualistic interactions with arbuscular mycorrhizal fungi (AMF), symbiotic fungi of almost 80% of land plants, which provide water and minerals (phosphate and nitrogen) in exchange of carbohydrates (Ho-Plágaro et al., 2020). Besides AMFs, some plant families, notably, legumes, establish symbiotic interactions with nitrogen-fixing bacteria (Bagyaraj, 1991; Ramasamy and Muthukumar, 2019). This property is of tremendous importance in the objective to reduce chemical fertilizers and reach sustainable agriculture (Ramasamy and Muthukumar, 2019).

However, the effect on the mutualistic interactions of the introduction in the rhizosphere of *P. oligandrum* and, more generally, of BCAs on the functioning of the root microbiota remained poorly understood. A study focusing on a fungal and oomycete population after the introduction of *P. oligandrum* into the rhizosphere of tomato plants concluded that *P. oligandrum* did not modify the microbial ecosystem (Vallance et al., 2009). More recently, it was found that wheat seed dressing with the fungal mycoparasite *Trichoderma atroviride* significantly modified the composition and structure of the fungal community (Sui et al., 2022). However, the specific effect of these BCAs on mutualistic interactions was not investigated.

In this context, our research aims at studying the potential impact of BCAs such as *P. oligandrum* on root–microbe interactions and the composition of the root microbiota. To address these points, we report here the establishment of a biological system involving *P. oligandrum* and two legume plants, namely, the model legume *Medicago truncatula* and the agronomic relevant legume, *Pisum sativum* (garden pea). We first dissected the effects of root inoculation with *P. oligandrum* on plant growth and immunity to evaluate the impact of *P. oligandrum* on protection against pathogenic attack, establishment of mutualistic interactions, and, more globally, on root microbiota.

## Materials and methods

### Microbial strains and culture conditions

The M1 (ATCC384722) strain of *P. oligandrum* was cultured and grown on V8 juice (Campbell’s, USA) 10% (v/v) supplemented with 2 g L^-1^ of CaCO_3_ and agar in 90-mm Petri dishes. Oospore collection was obtained through a culture of 7- to 14-days-old of *P. oligandrum* on solid V8 and by washing with 8–10 mL of distilled water, followed by filtration through 100-μm filters. The final concentration of oospores was adjusted so that each seedling received around 10,000 oospores per milliliter.

Mycelium production was carried out through a liquid culture of *P. oligandrum* obtained from adding 10 plugs of *P. oligandrum* to 100 mL of liquid V8 medium and maintaining the mixture in the dark at 28°C for 3 days in Roux flasks. The *P. oligandrum* mycelium was then washed with distilled water and filtered through 100-μm fiber filters. Finally, around 15 g of mycelium was then blended in 100 mL of distilled water for the inoculation solution.

*Aphanomyces euteiches* Drechsler isolates MF1, an alfalfa-infecting strain, and RB84, a pea-infecting strain, were grown and maintained on corn meal agar plates in the dark at 22°C (Malvick and Grau, 2001; Moussart et al., 2008). Zoospores were obtained based on the previously described protocol (Badreddine et al., 2008).

A rifampicin-resistant isolate of *Ensifer meliloti* CCMM B554 strain (Nagymihály et al., 2017) was transformed with the pHC60-GFP plasmid (Cheng and Walker, 1998; Cheng and Yao, 2004) by tri-parental mating. The resulting pHC60-GFP *E. meliloti* CCMM B554 strain was grown on tryptone yeast (TY) medium (Beringer, 1974) with the following modifications: pH was adjusted to 6.8, and TY medium was supplemented by adding CaCl_2_ at a final concentration of 20 mM after autoclaving. The strain and the plasmid were selected using rifampicin (100 µg mL^−1^) and tetracycline (5 µg mL^−1^), respectively. A pre-culture was inoculated by adding a loop of bacteria to 100 mL of liquid TY medium (pH = 6.8; CaCl_2_, 20 mM; rifampicin, 50 µg mL^−1^; tetracycline, 2 µg mL^−1^) and was grown at 28°C for 2 days in a shaker incubator (220 rpm). The cultures were then centrifuged at 5,000 rpm for 10 min, followed by two successive washes with 10 mL of sterile distilled water. The final OD used for plant inoculation was adjusted to 0.05 (10^8^ CFU mL^−1^). The GFP-transformed *E. meliloti* strain CCMM B554, also known as FSM-MA, was maintained on TY medium (for 1 L, 5 g of Difco Bacto tryptone and 3 g of Difco Bacto yeast extract, with the pH adjusted to 6.8), supplemented by 1 mL of CaCl_2_ (20 mM), and selected with rifampicin (100 µg mL^-1^) (Gage et al., 1996; Fox et al., 2011; Nagymihály et al., 2017). A pre-culture was done by adding a loop of bacteria to 100 mL of liquid TY media supplemented with liquid CaCO_3_ at 20 mM and antibiotics, including tetracycline 2 µg mL^-1^ and rifampicin 50 µg mL^-1^, and was kept at 28°C for 2 days in a shaker incubator. The cultures were then centrifuged at maximum speed for 10 min and followed by two successive washes with 10 mL of distilled water. The final OD for the applied solution was adjusted to 0.05 (10^8^ CFU mL^−1^).

*Rhizophagus irregularis* DAOM 197198 inoculum was a suspension of spores derived from CONNECTIS™ (Connectis AMM no. 150007, Agronutrition, Carbonne, France). The base solution was at 1,000 spores per milliliter, which was finally diluted to 500 spores per milliliter in the applied solution.

### Plant material, growth condition, and inoculation procedures

A17 and F83005.5 accessions of *M. truncatula* [Gaertn.] seeds were scarified in sulphuric acid for 5 min, washed three times in water, and then sterilized in 2.4% active chlorine bleach for 3 min before three washes in sterile water. To induce germination, seeds were soaked in water for 1 h for imbibition and then placed on 1% agarose in Petri dishes incubated at 22°C for 2 or 3 days in the dark.

The Cv. Precovil cultivar (Vilmorin, France) of *Pisum sativum* [L.] seeds were surface-sterilized and scarified by immersion in 70% ethanol (v/v) for 1 min, followed by 10 min of immersion in 2.4% active chlorine bleach. Subsequently, the seeds were washed with sterile water and left for pre-germination on water–agar 1% at 28°C for 5 days in the dark.

For plant growth stimulation assays with *P. oligandrum*, germinated *M. truncatula* A17 or *P. sativum* cv. Cv. Precovil seedlings were cultivated in each pot containing 1:1 v/v mixture of soil and sand. Then, 10 mL of *P. oligandrum* mycelium was added to pots of 300-mL capacity, while for 2-L round pots, 66.6 mL of mycelium containing 15 mg of mycelium in 100 mL of water was added (the amount of soil in the pots was approximately 300 mL and 2 L, respectively). The *Pythium* solution was added directly to the pots after planting the seedlings. The plants were then kept under controlled conditions in greenhouse (22°C, 14-h light photoperiod) or in phytotron (22°C, 80%, 16:8-h light/dark photoperiod). The nutrition solution was given to plants by watering every 5 weeks according to the manufacturer’s recommendation (“Engrais Universel Toute Plante”, Algoflash, France). For pea growth stimulation, a total of 10 plants with five replications per condition were tested, while for *M. truncatula* growth promotion test the total number of tested plants were 20, with 10 replications per condition. For plant protection assays with *P. oligandrum*, for *M. truncatula* 10 mL of MF1 and for *P. sativum* 10 mL of RB84 *A. euteiches* zoospore solution, with a final concentration of 25,000 zoospores per pot, were added directly to the seedlings using the same setup in the plant growth stimulation assays. The total number of *M. truncatula* plants in this experiment was 14, with seven biological replications per modality, while a total number of 10 plants with five replications per condition were considered for the pea protection assay.

For *in vitro* inoculation with *E. meliloti*, five A17 *M. truncatula* seedlings were placed on Fahraeus media (0.132 g L^-1^ CaCl_2_, 0.12 g L^-1^ MgSO_4_·7H_2_O, 0.1 g L^-1^ KH_2_PO_4_, 0.075 g L^-1^ Na_2_HPO_4_·2H_2_O, 5 mg L^-1^ NaFe EDTA, and 0.07 mg L^-1^ each of MnCl_2_·4H_2_O, CuSO_4_·5H_2_O, ZnCl_2_, H_3_BO_3_, and Na_2_MoO_4_·2H_2_O, adjusted to pH 7.5 before autoclaving, supplemented with 1.5% agar) on 12-cm^2^ petri dishes. In addition, 20 mL of *E. meliloti* mixture at OD of 0.05 (10^8^ CFU mL^−1^) was added to each Fahraeus plate with gentle stirring and was removed after 1 h. Around 10,000 *P. oligandrum* oospores were added to each seedling in each square petri dish. Seven biological replications were considered for each condition. The plates were kept at 22°C under 16-h light and 8-h dark photoperiod for 35 days.

For the experiments carried out in pots, we used sterilized vermiculite as substrate. Pregerminated A17 seedlings were placed in each pot, and then 10 mL of *E. meliloti* mixture with 0.05 OD (10^8^ CFU mL^−1^) was added to the pots near the seedlings. *P. oligandrum* mycelium solution containing 15 g of fresh mycelium in 100 mL of water was also added near the seedlings at the same time. Seven biological replications per condition were considered. Watering was carried out with both water and nutrient solution without nitrogen (N/P/K = 0/15/40, supplied by PlantProd®, ref. 211.00, Fertil S.A.S., Boulogne Billancourt, France), alternating two waterings with deionized water and one watering with Plant Prod solution (1 g L^-1^, pH 7).

Mycorrhization assays with *R. irregularis* were carried out by adding one A17 *M. truncatula* seedling to each pot filled with around 300 mL of a mixture consisting of 20% vermiculite and 80% zeolite (fine and coarse, 1:1 v/v). Before filling the pots, the fine zeolite was passed through a 300-µm sieve, while a 630-µm one was used for the coarse zeolite. Together with vermiculite, they were put in the oven for 5 h at 180°C. They were then mixed with 20% osmotic water. The *R. irregularis* spore solution concentration was adjusted to 500 spores mL^−1^, and 1 mL was added near the seedling in the substrate, while the *P. oligandrum* mycelium solution constituted 15 g of mycelium in 100 mL of water, which was likewise added near the seedling at the same time. For each condition, seven biological replications were considered.

### Transcriptomics

*P. oligandrum*-inoculated and non-inoculated A17 *M. truncatula* seedlings were grown on M medium prepared based on Becard and Fortin (1988). In the *P. oligandrum* treatments, each seedling received 10,000 oospores per milliliter. Petri dishes were then placed in a phytotron at 22°C under 16-h light and 8-h dark photoperiod. Total RNA comprised of a pool of five seedlings in each replication of different conditions, at 3, 5, 7, and 14 dpi, was then extracted by using RNeasy plant mini kit (Qiagen) according to the manufacturer’s instruction, and rough RNA purity was checked using NanoDrop (Thermo Fisher). The quality control, library preparation, and sequencing were based on the kit Illumina TruSeq Stranded mRNA, sequenced with high-throughput Illumina Novaseq (2×150 pb). The quality of the obtained sequence was verified by using FastQC. HTseq-counts files were analyzed with the R software, using also EdgeR package version 3.24.3 (McCarthy et al., 2012).

Genes which did not have at least one read after a count per million normalization in at least one half of the samples were discarded. Raw counts were normalized using TMM method, and count distribution was modeled with a negative binomial generalized linear model where the treatment, the time, and the double interaction between treatment and time were considered and where the dispersion is estimated by the EdgeR method (Robinson et al., 2009). A likelihood ratio test was performed to evaluate an infection effect at a given time point. Raw *p*-values were adjusted with the Benjamini–Hochberg procedure to control the false discovery rate (FDR). The raw files were imported to R software for data analysis (http://www.r-project.org/). After that, principal component analysis was done on normalized data. Differentially expressed genes (DEGs) were then selected based on FC>1 or FC<-1 (log2). Clustering took place by HCE3.0 software with DEGs average linkage (UPGMA) Euclidean distance. After that, gene ontology was done through ShinyGo visualization (Ge et al., 2020) and with Mapman analysis.

### Metagenomics

F83005.5 accession of *M. truncatula* seedlings was grown in pots containing a 1:1 v/v mixture of soil and sand and kept under controlled conditions in phytotron (22°C, 80%, 16:8-h light/dark photoperiod). Seven replications per condition were considered. At 56 days post-inoculation (dpi) (or 2 months after inoculation), the plants were uprooted and the rhizosphere was separated by reversing the pots, the whole plant was brought out of the pot. By a gentle shake, the loosened soil (bulk) was removed. The root was then cut from the aerial part and put in a 50-mL Falcon tube filled with 30 mL of sterile water. To separate the roots from the soil, a vortex at maximum speed was applied for about 2 min. The roots were then removed from the tube with the help of forceps. The Falcon tube was centrifuged for 30 min at maximum speed. The supernatant was then removed, and the pellet was put in Eppendorf tubes and kept at 20°C. The DNA was then extracted with a quick DNA fecal/soil microbe miniprep kit (Zymo Research Europe GmbH, Freiburg, Germany).

Libraries were generated using the Illumina two-step PCR protocol and normalized using SequalPrep plates (ThermoFischer™). The bacteria- and fungi-specific primer sets targeting the V3–V4 region of the small ribosomal RNA gene (16S rDNA) and ITS2 are presented in **Table 1**. Paired-end sequencing with a 2 × 250-bp read length was performed at the Bio-Environment platform (University of Perpignan Via Domitia Perpignan, France) on a MiSeq system (Illumina) using v2 chemistry according to the manufacturer’s protocol. The amplicon sequencing paired-end service on the Illumina MiSeq platform (2 × 250 bp) was performed by the Bio-Environment platform of Perpignan, France (plateformes.univ-perp.fr). Illumina sequence reads in the FastQ format were processed using QIIME2 (version 2021.4) (Bolyen et al., 2019). The DADA2 workflow (Callahan et al., 2016) was used to merge paired-end reads, perform filtering, dereplication, chimera removal, and identify representative sequences of amplicon sequence variants (ASVs). The taxonomic affiliations for the rRNA gene sequences 16S and ITS2 were, respectively, assigned using a pre-trained naive Bayes classifier on the basis of the SILVA 138 database (Quast et al., 2013) and the UNITE v8 database (Nilsson et al., 2019).

**Table 1:** Primers used in this study for 16S rDNA and ITS2 amplicon sequencing.

Application	Primers	Sequence (5′–>3′)	Annealing temperature (°C)	Reference
16S rDNA gene amplicon	Bact_341F	CCTACGGGNGGCWGCAG	65°C (30 cycles)	Herlemann et al., 2011
	Bact_805R	GACTACHVGGGTATCTAATCC		
ITS2 gene amplicon	ITS86F	GTGAATCATCGAATCTTTGAA	62°C (30 cycles)	(Op De Beeck et al., 2014)
	ITS4	TCCTCCGCTTATTGATATGC		

The sequencing data were processed under R v3.4 (www.r-project.org) using the R-Studio (http://www.rstudio.com/) Phyloseq package (McMurdie and Holmes, 2012). Beta diversity for all conditions was calculated using the Bray–Curtis dissimilarity and compared using analysis of similarities (ADONIS) on normalized data (999 permutations) through Vegan R packages (Oksanen et al., 2020). Kruskal–Wallis tests were performed to compare diversity and richness indices between the conditions. Determination of statistically significant differences (*p*-value < 0.01) in ASV abundance was performed using the DESeq2 package to compare the impact of *P. oligandrum* on the rhizosphere (Love et al., 2014).

### Metabolomics

The impact of *P. oligandrum* on medium-polar metabolites was tested within an *in vitro* system with five replications and three time points (3, 5, and 7 dpi) for inoculated and non-inoculated A17 *M. truncatula*. Five A17 *M. truncatula* seedlings were placed on M medium into 12-cm^2^ Petri dishes for each condition with six replications. Each seedling was inoculated with around 10,000 oospores per milliliter. They were then kept in a phytotron at 22°C under 16-h light and 8-h dark photoperiod. At the designated time points, medium-polar metabolites were extracted from the pool of five seedings for each replication of each condition based on Salem et al. (2016). The roots were put in 2-mL Eppendorf tubes accompanied with two steel beads of 4 mm in size and were ground with a Mixer Mill MM 400 grinder through two cycles of 30 s at 30 Hz (Retsch, Eragny sur Oise, France). Moreover, 2-mL FastPrep tubes (MP Biomedicals Lysing Matrix D, Illkirch, France) were filled with 80 mg of tissue powder containing 1.4-mm ceramic spheres and 700 µL of M1 solution (methyl tert-butyl ether/methanol, 3:1). After two cycles of 20 s at 6 m/s in FastPrep-24™ (MP Biomedicals™), 700 µL of M2 solution (MeOH/H_2_O, 1:3) was added, followed by 30 s of vortexing at maximum level. The tubes were then centrifuged at 4°C and 10,000 rpm for 20 min. All supernatants were then collected separately. Among the three observed phases, the lower phase containing medium-polar metabolites were used for ultra-high-pressure liquid chromatography–high-resolution mass spectrometry (UHPLC–HRMS). The non-polar phase was then evaporated overnight with SpeedVac™ SPD111V (ThermoFisher Scientific™), and then the normalized amount of MeOH/H_2_O (2 mg mL^-1^), based on the tubes’ weight before and after SpeedVac™, was added to each tube. Then, 0.2-μm PTFE filters (Thermo Scientific™) were then used to filter the extracts, and they were finally transferred into vials. The extraction blanks and quality control were also provided for extraction and analytical validation.

UHPLC–HRMS analyses were performed on a Q Exactive Plus quadrupole mass spectrometer, equipped with a heated electrospray probe (HESI II) coupled to an U-HPLC Vanquish system (ThermoFisher™ Scientific, Hemel Hempstead, UK). The samples were separated on a Luna Omega Polar C18 column (150 × 2.1 mm i.d., 1.6 μm, Phenomenex, Sartrouville, France) equipped with a guard column. Mobile phase A (MPA) was water with 0.05% formic acid (FA), and mobile phase B (MPB) was acetonitrile with 0.05% FA. The solvent gradient was as follows: 0 min, 98% MPA; 0.5 min, 98% MPA; 10.5 min, 70% MPB; 10.6 min, 98% MPB, 12.6 min, 98% MPB; 12.7 min, 98% MPA; and 14 min, 98% MPA. The flow rate was 0.4 mL/min, and the column temperature was adjusted to 40°C, while the autosampler temperature was fixed to 10°C. The injection volume was adjusted to 5 µL for root extracts. The mass parameters were used based on Fraisier-Vannier et al. (2020) and Chervin et al. (2022).

LC–MS data were processed by MS-DIAL 4.80 for mass signal extraction between 100 and 1,500 Da from 0.5 to 12 min (Tsugawa et al., 2015). MS1 and MS2 tolerance were set to 0.01 and 0.05 Da, respectively, in the centroid mode. The optimized detection threshold concerning MS1 for positive and negative mode was set to 1.5.10^6^ and 2.10^6^, respectively. Peak alignment was done according to Fraisier-Vannier et al. (2020). MS-DIAL data were then cleaned by using MS-CleanR based on Fraisier-Vannier et al. (2020). An in-house database constructed on MS-FINDER version 3.52 model was used for peak annotation (Tsugawa et al., 2016; Chervin et al., 2022). C, H, O, N, P, and S atoms were utilized principally in Formula finder. Databases based on *Medicago* (genus) and *Fabaceae* (family) and dictionary of natural product were constituted and used (DNP-CRC press, DNP on DVD v. 28.2). Through a successive ranking based on a genus database, then family database, and finally generic database, annotation prioritization was performed using the final MS-CleanR output. The generic databases were KNApSAcK, PlantCyc, and PubChem available within MS-FINDER.

Normalized LC–MS dataset created by using MS-CleanR was imported in R for multivariate data analysis. The mixOmics package (Rohart et al., 2017) was used to perform principal component analysis (PCA) as a first exploratory step before discriminant modeling. All data were centered and scaled to unit variance. To consider the effect of dpi, data were sloped between 3 to 7 dpi and 3 to 14 dpi. Partial Least-squares Determinant Analysis (PLS-DA) model was applied on this transformed dataset to extract features correlated to each sample class. The loading weights of each variable were extracted from the two first components to rank the top 50 loadings of the discriminant model. Finally, these 50 features (m/z × RT pairs) were used to construct a clustered image map by using euclidian distance and ward agglomeration for both rows (features) and columns (samples).

### Microscopy

For visualization and counting of vesicular and arbuscular structures formed by *R. irregularis* colonization, coloration with ink and vinegar was carried out (Vierheilig et al., 1998), and the grid intersect method was used to assess the frequency of mycorrhizal colonization events (Giovannetti and Mosse, 1980). For experiments using the *E. meliloti* GFP strain, all observations were done using a green fluorescent protein (Nikon P2-EFL, GFP-L) filter with an excitation filter of 460–500 nm, dichroic mirror of 505, and barrier filter of 510, resulting in a fluorescent green color. The microscopical observations were done by using a dissecting microscope, Nikon SMZ18, using BF, GFP-L, and RFP-B filters depending on the experiment.

### Statistics and data analysis

The general statistical analysis was done using SAS 9.4 software, and graphs were made by using Graphpad prism 8.0.2 software (San Diego, CA, USA). Transcriptomic and metagenomic data were analyzed using R software as well as EdgeR package versions 3.24.3 and R v3.4 (http://www.r-project.org/) using the R-Studio (http://www.rstudio.com/), respectively.

Statistical analyses for metabolomics were done using SIMCA (version 14.1, Umerics, Umea, Sweden). All data were scaled by unit variance scaling before the multivariate analysis. The partial least squares-discriminant analysis (PLS-DA) and orthogonal projection to latent structure using discriminant analysis was used to separate data according to *M. truncatula* growing conditions. The general statistics was performed using SAS 9.4 software.

## Results

### *Pythium oligandrum* promotes the growth and seed production of *M. truncatula* and *P. sativum* and protect against the root pathogen *A. euteiches*

**Figure 1:**
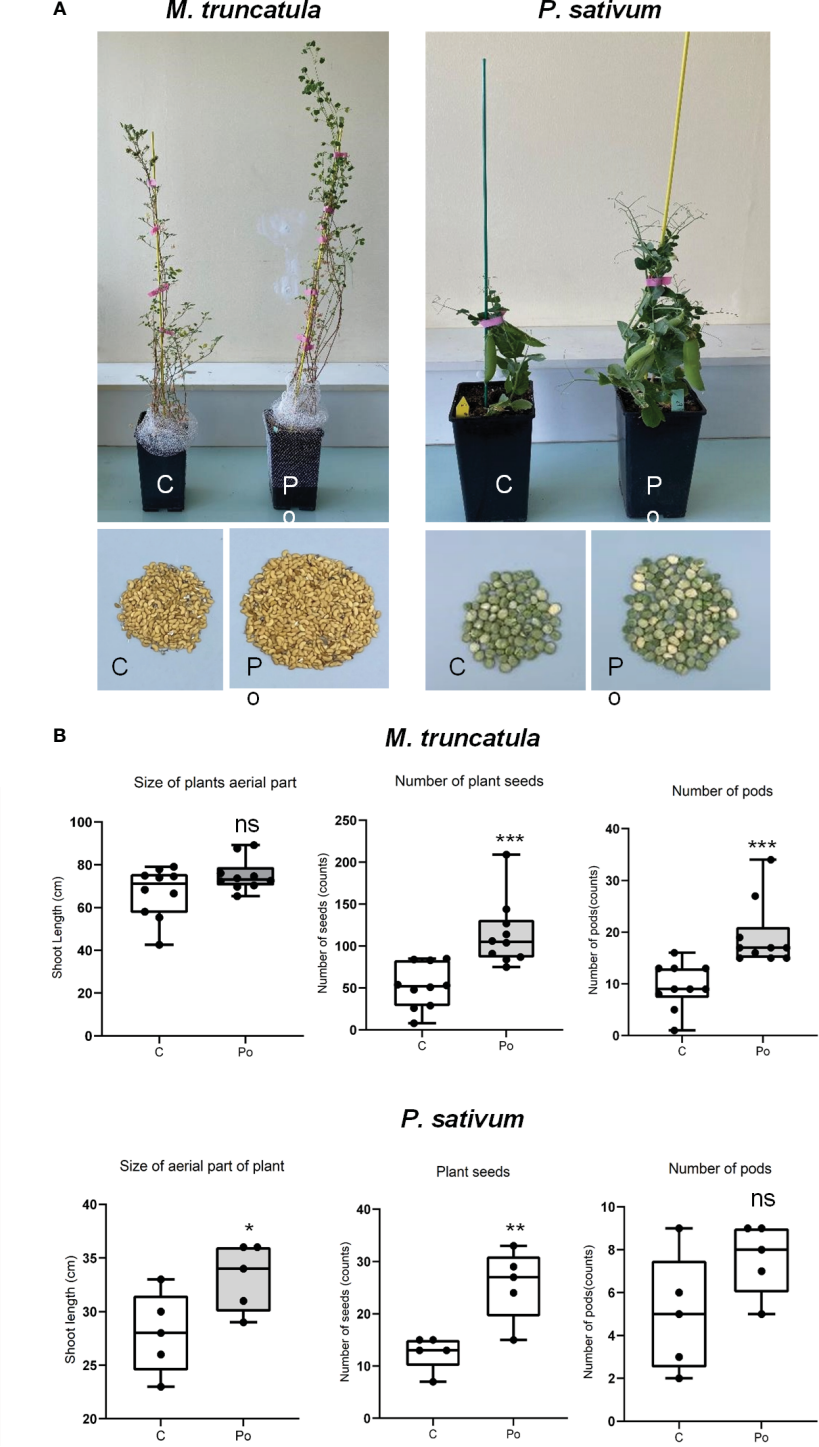
Comparison of different treatments on *M. truncatula* and *P. sativum* development and yield in soil. **(A)** A17 *M. truncatula* and *P. sativum* cv. Precovil plants at 90 and 42 days post-inoculation, respectively. **(B)** Box plots of shoot length, number of seeds, and number of pods in control and *P. oligandrum* M1 strain mycelium (Po) conditions; *n* = 10 for *M. truncatula* and *n* = 5 for *P. sativum*. *, **, and *** mean significance at 0.05, 0.01, and 0.001 probability level, respectively, and n.s. means not statistically significant based on Student’s *t*-test.

*Pythium oligandrum* was previously reported to promote plant growth and protect them from diseases in several plant species. Here we investigated the effects on legume plant development and on protection against the legume root pathogen *A. euteiches*. To do so, we developed a soil inoculation assay with the ground mycelium of *P. oligandrum* in which seeds of A17 *M. truncatula* and *P. sativum* Cv. Precovil line were sown (**Figure 1A**). A significant positive effect of *P. oligandrum* was observed both on plant height at 42 days post-inoculation and on the number of seeds formed by plants (**Figure 1B**). *M. truncatula* did not show a significantly enhanced growth but developed more pods and seeds at 90 days post-inoculation with *P. oligandrum* (**Figure 1B**), whereas *P. oligandrum* increased the size of pea plants and the number of seeds produced but not the number of pods.

**Figure 2:**
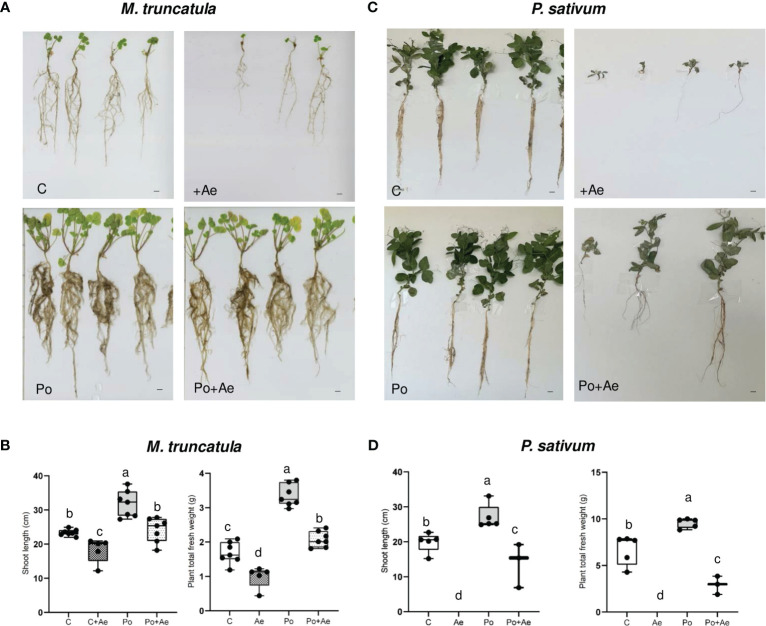
Effect of *P. oligandrum* on pea and *M. truncatula* growth and protection against root rot causal agent *A*. *euteiches*. **(A)** Images of representative *M. truncatula* F83005.5 seedlings at 40 days post-inoculation (scale bars = 2 cm). **(B)** Shoot length and plant fresh weight of *M. truncatula* F83005.5 in control non-inoculated **(C)**, *A*. *euteiches* (Ae), *P. oligandrum* M1 strain mycelium (Po), and *P. oligandrum* + *A. euteiches* (Po + Ae) conditions. **(C)** Images of representative *Pisum sativum* cv. Precovil plants at 28 days post-inoculation (scale bars = 2 cm). **(D)** Shoot length and plant fresh weight of *Pisum sativum* cv. Precovil in control non-inoculated **(C)**, *A*. *euteiches* (Ae), *P. oligandrum* M1 strain mycelium (Po), and *P. oligandrum* + *A. euteiches* (Po + Ae) conditions. The mean of each trait for each condition is compared with a Duncan test, and means labeled with the same letter are not statistically significant.

We then assessed whether *P. oligandrum* may protect *P. sativum* and *M. truncatula* against the root rot caused by the legume pathogen *A. euteiches*. We first inoculated the highly susceptible line *M. truncatula* F83005.5 line with the alfalfa isolate MF1 of *A. euteiches*. As shown in **Figures 2A, B**, all plants treated with *A. euteiches* showed a clear reduction of disease symptoms and displayed a statistically inferior mass of aerial part and whole plants as compared to control plants. Plants treated with *P. oligandrum* again showed enhanced growth as compared to the control.

Inoculation with *A. euteiches* pea isolate RB84 on *P. sativum* cv. Precovil resulted in a severe reduction of plant aerial size and total plant weight (**Figures 2C, D**) as compared to the control plant. Similar to *M. truncatula*, *P. oligandrum* plants showed enhanced biomass, and inoculation with both *P. oligandrum* and RB84 slightly reduced the detrimental effect on shoot length and plant weight as observed following *A. euteiches* inoculation.

### *Pythium oligandrum* strongly activates *M. truncatula* defense responses and isoflavonoid metabolism

**Figure 3:**
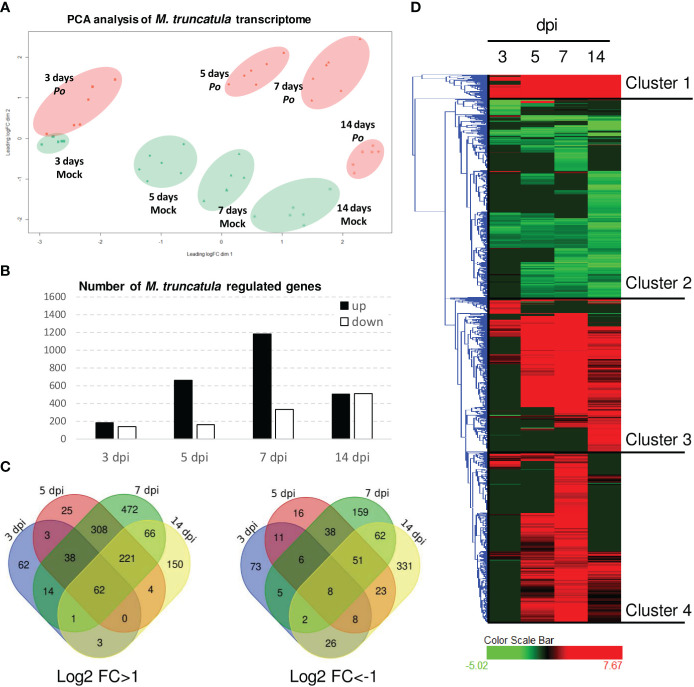
Transcriptomic analysis of *Pythium oligandrum*-inoculated roots. RNAseq experiments were performed on RNA extracted from *in vitro* grown plants inoculated with *P. oligandrum* oospores at different time points. **(A)** Principal component analysis of data obtained from the RNA sequencing of *P. oligandrum* and A17 *M. truncatula* at 3, 5, 7, and 14 days post-inoculation. Orange dots show data belonging to *M. truncatula* plants inoculated with *P. oligandrum*, and green dots show data belonging to mock plants. **(B)** Number of differentially expressed genes (DEGs) at each time point. **(C)** Venn diagrams representing the DEGs for each condition. **(D)** Hierarchical clustering on DEGs; clustering was obtained using Hierachical Clustering Explorer version 3.5 with default parameters.

In order to better understand the molecular basis of plant growth promotion and protection caused by *P. oligandrum*, we probed A17 *M. truncatula* responses to *P. oligandrum* oospores with transcriptome and metabolome analyses within an *in vitro* culture system at 3, 5, 7, and 14 dpi. These time points were chosen to observe the early responses of root colonization by *P. oligandrum*. For transcriptomic studies, after RNA sequencing and mapping on model genes (**Supplementary Material S1**), we performed a principal component analysis (**Figure 3A**). The difference between mock and *P. oligandrum* datasets could be observed as soon as day 3 post-inoculation but was more noticeable at 5 and 7 days post-inoculation, and then it tends to decrease at 14 days. The fold changes of each individual gene were calculated relatively to the control plants for each time point. In total, 2,236 differentially expressed genes (log_2_ FC > 1 or FC < -1; FDR < 0.01 in at least one condition) were obtained and subjected to further analysis. The number of induced and repressed genes are presented in **Figure 3B**. The strongest transcriptional response was observed at 7 dpi, where 1,182 genes were induced by the oomycete (**Figures 3B, C**). Clustering analysis allowed the definition of four well-defined clusters among the 2,236 DEGs (**Figure 3D**). Cluster 1 represented genes strongly induced at all time points of the kinetic, cluster 2 was the group of genes being repressed at one or several time points, cluster 3 represented genes induced in later time points of the kinetic (5, 7, and 14 dpi), and cluster 4 was the group of genes mostly induced at 5 and 7 dpi (**Figure 3C**).

**Figure 4:**
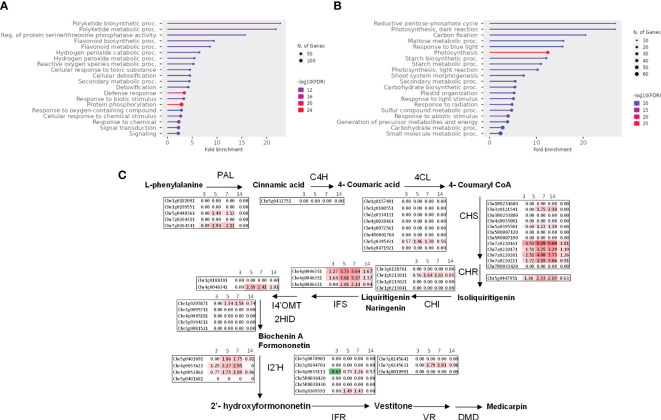
*Pythium oligandrum* root colonization induces *M. truncatula* defense genes and the expression of the isoflavonoid pathway. **(A)** Upregulated genes (clusters 1, 3, and 4) and **(B)** downregulated genes (cluster 2) were analyzed using ShinyGO with the GO parameter “Biological Process”. **(C)** Log_2_ fold change of the expression of genes coding the biosynthetic enzymes of medicarpin at 3 to 14 dpi with *P. oligandrum.* The biosynthetic pathway for medicarpin and related isoflavonoids is shown (Naoumkina et al., 2006) for each time point as indicated. PAL, L-phenylalanine ammonia-lyase; C4H, cinnamate 4-hydroxylase; 4CL, 4-coumarate:CoA ligase; CHS, chalcone synthase; CHI, chalcone isomerase; IFS, isoflavone synthase; I4′OMT, 2-hydroxyisoflavanone 4′-O-methyltransferase; 2HID, 2-hydroxyisoflavanone dehydratase; I2′H, isoflavone 2′-hydroxylase; IFR, isoflavone reductase; VR, vestitone reductase; DMID, 7,2′-dihydroxy-4′-methoxy-isoflavonol dehydratase.

We subjected the induced genes (clusters 1, 3, and 4) to gene ontology GO terms enrichment analysis using ShinyGO (Ge et al., 2020). Several GO terms related to the synthesis of isoflavonoids, a major class of antimicrobial compounds in *Medicago* species (Gholami et al., 2014), were found (**Figure 4A**). Downregulated genes fall mainly in GO categories belonging to primary metabolism such as the reductive pentose-phosphate cycle (**Figure 4B**). Strikingly, enzymes involved in the metabolism of isoflavonoids were strongly upregulated notably at 7 dpi (**Figure 4C**).

**Figure 5:**
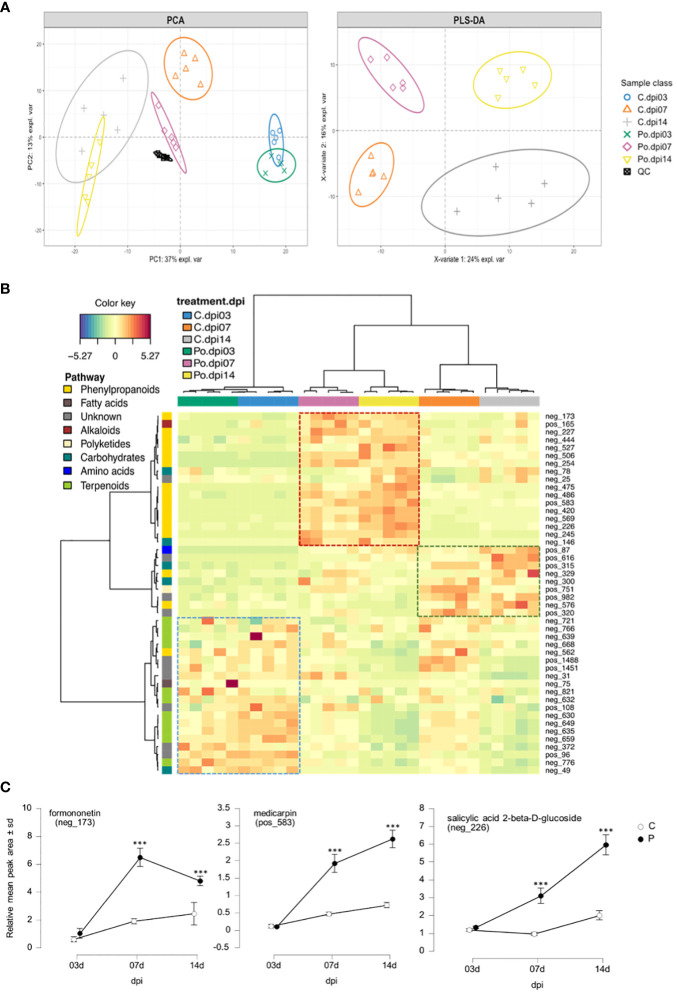
Medicarpin and formononetin are two major components of *Medicago truncatula* metabolic response to *Pythium oligandrum*. **(A)** Principal component analysis score plot of LC–MS dataset from PC1 and PC2, and PLS-DA score plot from the two first latent variables after slope-based transformation. Data were centered and unit scaled. **(B)** Clustered Image Map from the most important variables selected by PLS-DA model. Rows display the LC–MS features numbered according to **Supplementary Material S2**. Each feature is color-coded along the vertical axis according to the pathway of origin deciphered by NPclassifier. Column display samples. Each sample is color-coded according to its class (3, 7, or 14 dpi). Both rows and columns were clustered using Euclidian distance and ward agglomeration. The dotted line square displays the most representative cluster of each group. **(C)** Line plots of selected features from the top 50 PLS-DA loading weights. The data represent the relative mean peak area (Y) of each sample class along the dpi (X). The plotted features were selected for their high confidence annotation level from level 2 to level 3 in the genus or family. *** means significance at 0.001 probability level based on Student's T-test.

To confirm the induction of isoflavonoid biosynthesis by *M. truncatula* in response to *P. oligandrum*, we performed an analysis of *M. truncatula* root metabolome upon *in vitro* inoculation with the oomycete *P. oligandrum* oospore M1 strain through 3, 7, and 14 days post-inoculation kinetics. Based on retention time and mass spectra, major peaks were annotated and assigned through a comparison with standards and data from the literature sources. Mass spectrometry was carried out in positive and negative ionization modes. The total number of ions retained following MS-CleanR sorting was 474 (**Supplementary Material S2**). A global metabolomic analysis based on PCA validated our dataset by regrouping the samples according to their group of origin (**Figure 5A**). Then, a supervised PLS-DA resulted in the clear separation of control and *P. oligandrum*-treated plants at 7 and 14 dpi (**Figure 5A**). We then plotted the 46 most significant variables which supported the PLS-DA separation of the groups (**Figure 5B**) (**Supplementary Material S2**). The annotation of these variables revealed that salicylic acid 2-beta-D-glucoside (variable neg_226) was the most strongly accumulated in *M. truncatula* roots at 7 and 14 days post-inoculation with *P. oligandrum* (**Figure 5C**); a similar trend was observed for the phenylpropanoids pterosupin (neg_420) and 3-hydroxy-8,9-dimethoxycoumestan (neg_245). Consistently with transcriptomic data, we found that *P. oligandrum*-inoculated plants strongly induced the accumulation of isoflavonoids such as medicarpin and formononetin at 7 and 14 dpi in the presence of the oomycete (**Figure 5C**). These transcriptomic and metabolomics data together support the role of *P. oligandrum* in root defense stimulation and notably the synthesis of antimicrobial isoflavonoids and phenylpropanoids.

### Root colonization by *P. oligandrum* does not hamper symbiotic interactions

As we documented a strong induction of plant defense mechanisms by *P. oligandrum*, we wondered whether root colonization by *P. oligandrum* could have a negative impact on the formation of symbiotic interactions such as nitrogen-fixing symbiosis and arbuscular mycorrhizal symbiosis. To study the nitrogen-fixing symbiosis, a biological system has been devised to study a possible interplay between *P. oligandrum* and GFP-tagged *E. meliloti* on A17 *M. truncatula*, during both early-stage and fully formed symbiosis.

**Figure 6:**
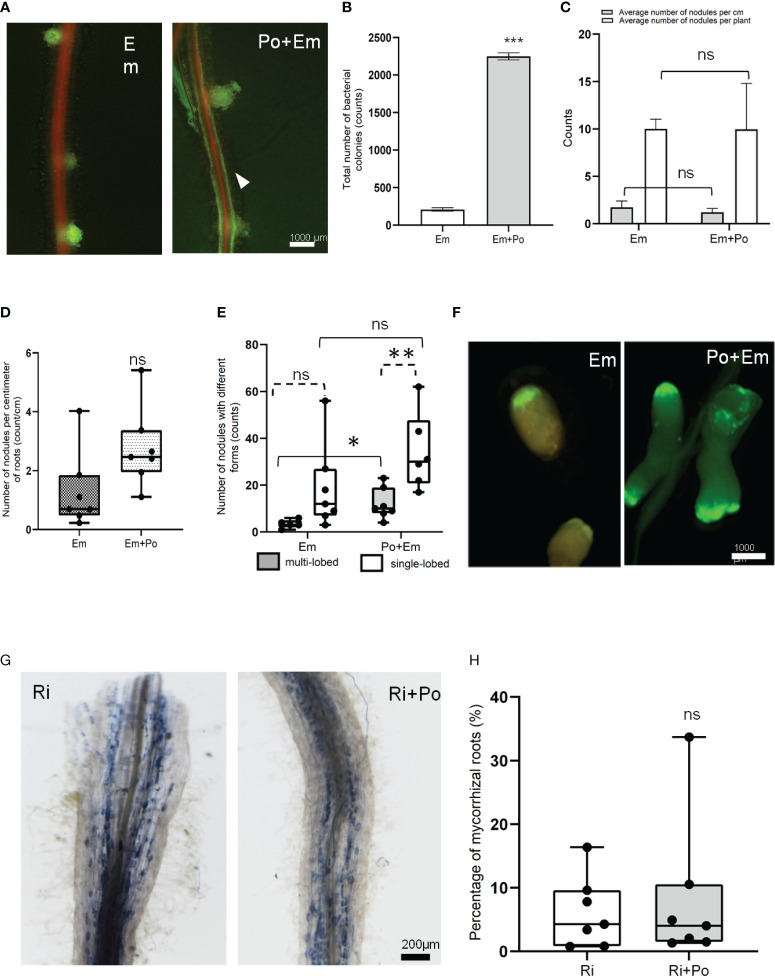
Effect of *P. oligandrum* on nitrogen fixing and arbsuscular mycorrhizal symbiosis in *M. truncatula*. **(A)** Representative *M. truncatula* A17 nodules with or without *P. oligandrum* inoculum. The arrowhead points to GFP-tagged *E*. *meliloti* biofilm formation around the *M. truncatula* A17 root in *P. oligandrum* M1 strain mycelium + *E*. *meliloti* (Po + Em) condition. **(B)** Counting of colony-forming units of *E*. *meliloti* inoculated alone on *M. truncatula* A17 (Em) or co-inoculated with *P. oligandrum* M1 strain mycelium + (Po + Em) at 35 days post-inoculation (dpi). **(C)** Counting of *M. truncatula* A17 nodules in Em and Po + Em at 35 dpi. **(D)** Box plots of the number of nodules per centimeter of *M. truncatula* A17 roots and **(E)** number of nodules with single- or multi-lobed shape upon inoculation with a GFP-tagged *E*. *meliloti strain* (Em) or *P. oligandrum* M1 strain mycelium + *E*. *meliloti* (Po + Em). **(F)** Images of representative nodules at 42 dpi. **(G)** Images of representative mycorrhizal roots inoculated or not with *P. oligandrum* M1 mycelium (Ri or Ri + Po). **(H)** Histograms of percentage of mycorrhized root in different conditions including *R. irregularis* (Ri) and *P. oligandrum* M1 strain mycelium + *R. irregularis* (Po + Ri) at 42 dpi. *** means significance at 0.001 probability, and n.s. means not statistically significant based on Student’s *t*-test. * and ** mean significance at 0.05 and 0.01, probability level, respectively, and n.s. means not statistically significant based on Student’s T-test.

Microscopic inspections of seedlings grown *in vitro* at 35 days post-inoculation showed that *P. oligandrum* did not affect the early stage of nodule establishment (**Figure 6A**). Relatively, we observed a brighter GFP signal all around the roots in the *P. oligandrum* conditions (**Figure 6A**). To confirm the stronger bacterial development around roots colonized by the oomycete, we collected the bacteria from each individual plate and performed a colony-forming unit count. This analysis confirmed that *E. meliloti* developed 10 times more (2.247 × 10^9^ CFU mL^-1^ in Po + Em condition *versus* 0.208 × 10^9^ CFU mL^-1^ in Em condition) around A17 roots when they were colonized by *P. oligandrum* (**Figure 6B**). Finally, in this *in vitro* assay, a similar amount of nodule formation was observed in the presence or absence of *P. oligandrum* (**Figure 6C**).

To assess the effect of *P. oligandrum* on the later stages of symbiosis, we performed experiments in pots. The mean number of nodules formed per centimeter of roots was similar in the two conditions (**Figure 6D**). However, while 11% of the nodules formed in the control situation displayed a multi-lobed morphology, this frequency increased to 29% in the *P. oligandrum*-treated situation (**Figures 6E, F**). This suggests that the *M. truncatula* responses to *P. oligandrum* may increase the development of multi-lobed nodules.

Regarding the effect of *P. oligandrum* on the establishment of arbuscular mycorrhizal (AM) symbiosis, we devised a co-inoculation assay in pots filled with A17 *M. truncatula*. Our results demonstrated that *P. oligandrum* does not inhibit mycorrhization in *M. truncatula* roots, as mycorrhizal structures including vesicles and arbuscular were detected in inoculated roots visualized by coloration and microscopy (**Figure 6G**). The rate of mycorrhization in the presence of *P. oligandrum* was indistinguishable from the control mycorrhizal inoculation (**Figure 6H**). Thus, the plant defense-stimulating and mycoparasitic *P. oligandrum* oomycete M1 strain does not affect the AM symbiotic interaction.

### *Pythium oligandrum* influences the composition of the rhizosphere microbiota

We then investigated the effect of root colonization by *P. oligandrum* on the rhizosphere of *M. truncatula* (F83005.5) plants sown in potting soil. A total of 14 samples comprising seven controls and seven *P. oligandrum*-inoculated plants were harvested at day 56 post-sowing, and DNA from their rhizosphere was extracted and subjected to PCR. The 16S rRNA dataset retained 194,708 reads, and the Internal Transcribed Spacer dataset retained 422,171 reads, which were assigned respectively to 1,413 bacterial amplicon single variants and 374 fungal ASVs (**Supplementary Materials S3**, **S4**). The data were normalized to account for unequal sequencing depth, resulting in a minimum sampling depth of 3,834 for 16S and 10,556 sequences per sample for ITS.

**Figure 7:**
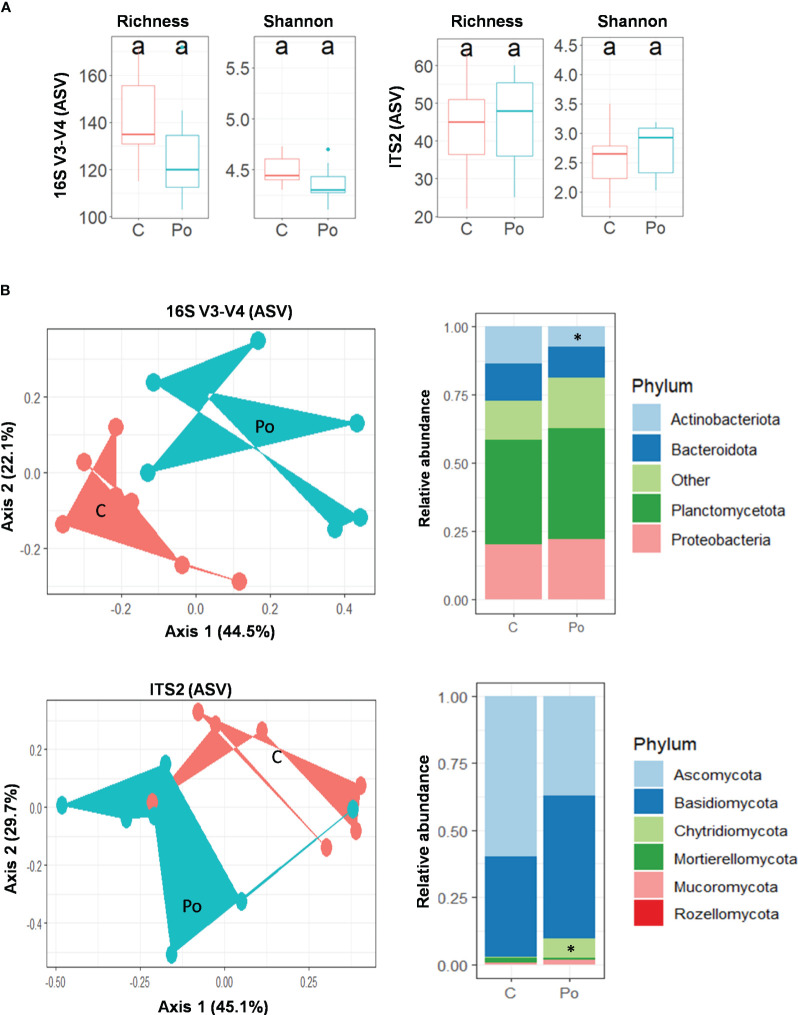
*Pythium oligandrum* shapes the bacterial and fungal communities associated to the rhizosphere of *Medicago truncatula* F83005.5 rhizosphere. **(A)** Alpha diversity measurements are based on observed ASVs and Shannon index for the bacterial and fungal microbiome. **(B)** Principal component analysis for beta diversity using Bray–Curtis distances in bacterial and fungal communities. C stands for control and Po for *P. oligandrum* M1 mycelium-inoculated plants. Relative bacterial and fungal abundances at phylum level. Statistical data analyses were performed using non-parametric Wilcoxon test (*p < 0.05).

The analysis of alpha diversity of bacteria and fungi (observed and Shannon diversity) in the rhizosphere of *M. truncatula* inoculated with *P. oligandrum* showed no significant difference *versus* the uninoculated control (**Figure 7A**). Conversely, the addition of *P. oligandrum* had a tremendous impact on the community structure (beta diversity) in both bacteria and fungi (**Figure 7B**). A principal component analysis (PCoA) based on Bray–Curtis dissimilarities revealed that the introduction of *P. oligandrum* is a large source of variation capturing 44.5% (PCoA, axis 1) of the variation of bacterial community composition and 45.1% (PCoA, axis 1) of the variation of fungal community composition (**Figure 7B**). The PERMANOVA tests confirmed a significant clustering of the inoculated sample and the uninoculated control in both bacteria (df = 1, *F* = 4.09, *r* ^2 ^= 0.25, *P* = 0.01**) and fungi (df = 1, *F* = 2.8, *r* ^2 ^= 0.19, *P* = 0.043*). The taxonomic assignment of ASVs suggested that four bacterial phyla, including Planctomycetota, Proteobacteria, Bacteroidota, and Actinobacteriota, dominated the rhizosphere regardless of the treatment. The pairwise comparison of relative abundances for each bacterial phylum showed that there was a significant drop in the phylum Actinobacteria (Wilcoxon: *p* > 0.05) among the bacterial community in the presence of *P. oligandrum*. The fungal taxonomic assignment showed the dominance of Ascomycota and Basidiomycota phyla in the non-inoculated control, whereas a third dominant phyla was detected in the rhizosphere of *P. oligandrum*-treated plants (Chytridiomycota; **Figure 7B**). Consistently, the pairwise comparison of relative abundances of fungal phyla showed that there was a significant increase in the phylum Chytridiomycota (Wilcoxon: *p* > 0.05) among the fungal community upon *P. oligandrum* treatment.

In order to determine more precisely whether particular bacterial or fungal genera are affected by *P. oligandrum* in their rhizospheric abundance, we performed a DEseq2 analysis on the individual ASVs of the dataset. A total of 17 bacterial ASVs and 20 fungal ASVs were differentially occurring upon *P. oligandrum* inoculation. We confirmed that *P. oligandrum* largely reduced rhizosphere colonization by the *Streptomyces*-related ASV219 belonging to Actinobacteria and found out that it impacts either positively or negatively 10 ASVs belonging to Planctomycota and four ASVs affected to Alphaproteobacteria (**Supplementary Material S5**). In addition, the oomycete inoculation increased the abundance of nine fungal ASVs related to Basidiomycota, among which six were belonging to the *Clitopilus* genus. Similarly, five Ascomycota-related ASVs were more abundant in the presence of the oomycete, and two showed the opposite behavior (**Supplementary Material S6**). Consistently with the PCoA, the Chytridiomycota ASV117 was enriched in *P. oligandrum* conditions by a fold change of 12.12. Taken together, these data illustrate how *P. oligandrum* can shape the host plant microbiota in addition to its impact on pathogenic interactions.

## Discussion

While *P. oligandrum* potential in crop protection has been largely documented, in the present study we aimed to describe the effect of oomycete on symbiotic interactions and microbial community. To do so, we devised biological systems with the model legume *M. truncatula* or pea and the oomycete.

Firstly, we validated that treatment of soil with *P. oligandrum* mycelium resulted in a higher yield and enhanced the growth in both *M. truncatula* and *P. sativum*. These findings are in line with previous reports which reported the same effect on cucumber (Kratka et al., 1994; Wulff et al., 1998). It is proposed that improved nutrient uptake by plant in the presence of *P. oligandrum*, notably phosphorus, gives rise to boosted plant growth (Kratka et al., 1994; Lugtenberg and Kamilova, 2009; Backer et al., 2018).

Secondly, we observed the efficacy of *P. oligandrum* treatment on legume plants’ protection against *A. euteiches*, an oomycete causing damping off and root rot, in *M. truncatula* and *P. sativum*. The ability of *P. oligandrum* to promote plant growth and nutrition may account for this improved resistance to the disease. In addition, the production of elicitors such as POD1 and POD2 cell wall proteins or of oligandrin by *P. oligandrum* can activate jasmonate and ethylene signaling; the oomycete can trigger host plant ferroptosis as well (Picard et al., 2000; Takenaka et al., 2003; Hase et al., 2006; Hase et al., 2008; Takenaka, 2015; Cheng et al., 2022). Collectively, these different mechanisms may account for the plant protection against *A. euteiches.* In line with this hypothesis, our transcriptomic data showed induction of defense genes, including secondary metabolites—notably, flavonoids. Several lines of evidence display the inhibitory potential of isoflavonoids against some pathogens within an *in planta* system (Stevenson et al., 1997). Previous work showed that rapid root colonization by *P. oligandrum* ends up with the activation of defense reactions through the phenylpropanoid pathway, which results in the production of secondary metabolites and formation of cell wall appositions (Benhamou et al., 1997; Benhamou et al., 2012; Bělonožníková et al., 2022). Our metabolomic data likewise proved the induction of flavonoid-like medicarpin and formononetin, two isoflavonoids with proven antifungal activity (Blount et al., 1992; Gupta et al., 2022). Accordingly, we have previously shown that this pathway is an essential component of the resistance of *M. truncatula* to *A. euteiches* (Badis et al., 2015).

Once we validated the effect of *P. oligandrum* on legume plants’ growth and protection, we decided to investigate its effect on mutualistic interactions, including the legume-specific nitrogen-fixing symbiosis. Interestingly, our results regarding the co-inoculation of *P. oligandrum* and *E. meliloti* showed that the number of *E. meliloti* colonies forming around the roots increased with the presence of *P. oligandrum*. This effect can be due to the stimulation by *P. oligandrum* of the flavonoid metabolism which has been shown to have a positive chemotaxis effect on rhizobia (Aguilar et al., 1988). In addition, the number of nodules formed in the presence of *P. oligandrum* was similar to that of the control, suggesting that the oomycetes do not compromise the symbiosis.

Regarding AMFs, a similar rate of mycorrhization was obtained between the control plants and *P. oligandrum*-treated plants. This suggests that the strong stimulation by *P. oligandrum* of various defense reactions (PR proteins and secondary metabolism) does not impair the interaction between plant roots and AMFs and that, in some way, AMFs are resistant to the mycoparasitic activity. Consistently, the isoflavonoids formononetin and biochanin A were formerly reported to improve the root colonization and hyphal growth of vesicular–arbuscular mycorrhizal bacteria ending in the stimulated growth of clover (Elias and Safir, 1987; Nair et al., 1991; Siqueira et al., 1991). Thus, stimulation of isoflavonoid biosynthesis by *P. oligandrum* may not hamper *M. truncatula* mycorrhization. Regarding arbuscular mycorrhizal symbiosis, a similar rate of mycorrhization was obtained between the control plants and the *P. oligandrum*-treated plants. This suggests that the strong stimulation by *P. oligandrum* of various defense reactions (PR proteins and secondary metabolism) did not impair the interaction between the plant roots and AMFs and that, in some ways, AMFs are resistant to the mycoparasitic activity. Consistently, the isoflavonoid formononetin and biochanin A were formerly reported to improve the root colonization and hyphal growth of vesicular–arbuscular mycorrhizal bacteria ending in the stimulated growth of clover (Elias and Safir, 1987; Nair et al., 1991; Siqueira et al., 1991). Thus, stimulation of isoflavonoid biosynthesis by *P. oligandrum* may not hamper *M. truncatula* mycorrhization.

Finally, we investigated whether *P. oligandrum* could have a wider impact on the microbial community of the rhizosphere. The analysis of alpha diversity showed no significant differences with the untreated control. However, the introduction of oomycete did change the community structure of both fungi and bacteria. These shifts in fungal and bacterial beta diversity has been documented previously and associated with beneficial outcomes (Vallance et al., 2012; Gerbore et al., 2014; Cernava et al., 2019; Uhlig et al., 2021).

In line with the drop of Actinobacteria abundance at the genera level in the presence of *P. oligandrum*, we observed a reduction in *Streptomyces* sp. ASV219. *Streptomyces* are major producers of antibiotics and antifungal, hence capable of inhibiting the growth of plant pathogens (Lee et al., 2018). Furthermore, they are considered as plant growth-promoting rhizobacteria involved in various activities such as phosphate solubilization, siderophore production, and nitrogen fixation (Bhatti et al., 2017; Rey and Dumas, 2017; Boubekri et al., 2022). Thus, the fact that *P. oligandrum* can modulate *Streptomyces* population in the rhizosphere may have important consequences on the microbiota functions. Regarding fungal communities, the result suggests that the mycoparasite *P. oligandrum* has a predation preference for Ascomycota-related ASVs rather than Basidiomycota. The *Trichoderma*-related ASV505 was still enhanced in the presence of the oomycete. *Trichoderma* are the most widely used fungal biocontrol agents against fungal diseases of pulses, grapes, cotton, onion, carrot, peas, plums, maize, apple, etc. (Kumar and Ashraf, 2017). From *in vitro* interaction assays of *P. oligandrum* and *T. harzianum*, it was concluded that the fungus can kill the oomycete (Floch et al., 2009). Thus, *Trichoderma*-related fungi may be enriched in the rhizosphere and use the oomycete as a prey. Their enrichment in presence of *P. oligandrum* may still participate in the protection against soil pathogens. Similarly, we noticed an enrichment of six ASVs belonging to *Clitopilus*, having some species considered as mycoparasitic basidiomycetes (Jatuwong et al., 2017). Finally, the enrichment of Chytridiomycota in response to *P. oligandrum* may participate in the control of insect populations since these fungi are often entomopathogenic (Kaczmarek and Bogus, 2021). Taken together, our data provide novel insights regarding the effect of *P. oligandrum* on the microbial communities of the rhizosphere and suggest that the oomycete may favor the recruitment of plant-beneficial microorganisms.

## Conclusion

The present study concluded that *P. oligandrum* is a potential biocontrol agent for legume crops, which could be used to promote plant growth and protect them from pathogenic attacks. We also revealed that *P. oligandrum* does not inhibit symbiotic interactions with other mutualistic microorganisms, including *E. meliloti* and *R. irregularis*. This beneficial mycoparasite likewise modifies the microbial community of the soil, and the functional outcome of these perturbations still needs to be investigated to address whether it benefits the plant by reducing or eliminating plant bacterial and fungal pathogens or by activating other mycoparasitic organisms that can fight against pathogens. Finally, the relative contribution in the protection against pests of *P. oligandrum* mycoparasitic *versus* plant defense-stimulating activities still has to be disentangled. The proven advantages of *P. oligandrum* still make it a great candidate for further studies in biocontrol industry and sustainable agriculture.

## Data availability statement

The sequence data were deposited at the Sequence Read Archive of the National Center for Biotechnology Information (NCBI) and are available under accession numbers PRJNA787426 and PRJNA787434 for 16S rDNA and ITS metagenomic data respectively. The RNA sequencing data of *M. truncatula* can be uploaded from the NCBI BioProject PRJNA806852.

## Author contributions

BD, EG, TR, and MH conceived this study. J-MC provided *E. meliloti* strain and information regarding nitrogen-fixing symbiosis assays. SR provided Connectis™ and information regarding mycorrhization assays. MH and RP coordinated the experiments and analyzed phenotypic data. GM and AA performed the metabolomics analysis. HS and MA analyzed the transcriptomic data, and KA and MZ analyzed the metagenomic data. MH along with BD, TR, and EG drafted the manuscript. All authors contributed to the article and approved the submitted version.
